# Short-Term Diet and Moderate Exercise in Young Overweight Men Modulate Cardiocyte and Hepatocarcinoma Survival by Oxidative Stress

**DOI:** 10.1155/2014/131024

**Published:** 2014-08-13

**Authors:** Marcellino Monda, Giovanni Messina, Ilaria Scognamiglio, Angela Lombardi, Giuseppe A. Martin, Pasquale Sperlongano, Marina Porcelli, Michele Caraglia, Paola Stiuso

**Affiliations:** ^1^Department of Experimental Medicine, Second University of Naples, 80138 Naples, Italy; ^2^Department of Biochemistry, Biophysics and General Pathology, Second University of Naples, 80138 Naples, Italy; ^3^Department of Veterinary Medicine and Animal Reproduction, Federico II University, 80138 Naples, Italy; ^4^Unit of General and Geriatric Surgery, School of Medicine, Second University of Naples, 80137 Naples, Italy

## Abstract

The present study was designed to evaluate the effects of diet lifestyle on extending lifespan and reducing liver cancer risk. Young overweight men (*n* = 20), without metabolic syndrome, were placed in a 3-week residential program on a low-fat diet and moderate aerobic exercise. In each subject, pre- and postintervention fasting blood were collected for evaluating levels of serum lipids, and oxidative stress markers. Using subject sera and cardiomyocyte (H9C2) culture systems, we measured heat shock protein 27 and 90 expression, lipid accumulation, and oxidative stress marker levels. After 3-weeks of diet, significant reductions (*P* < 0.05) in body mass index, serum lipids and lipid ratios, and oxidative markers were recorded. *In vitro*, we observed that the addition of postintervention sera increased H9C2 cell number and reduced HSP27 and 90 expression, mitochondrial superoxide anion, and lipid accumulation with a parallel increase in nitric oxide (NO) production (all *P* < 0.01). At the same time, postintervention sera decreased human liver hepatocellular carcinoma cell line (HepG-2) proliferation, lipid accumulation, oxidative stress, and extracellular-signal-regulated kinases (ERK1/2) activity. Lifestyle modification in young overweight men, without metabolic syndrome, could ameliorate cardiocyte survival and reduce hepatocellular carcinoma cell proliferation.

## 1. Introduction

Overweight, obesity, and inactivity are major risk factors for diseases such as type 2 diabetes, coronary heart disease, sleep apnea, cancer, and liver disease. Several lifestyle factors have been identified which may potentially counteract the negative health effects of obesity with the diet restriction (DR) and physical activity/exercise (PA). Diet restriction (including caloric restriction) is defined as a decrease in energy intake without lowering nutritional value. This simple intervention has shown, in a wide range of laboratory animals, to extend lifespan and decrease the incidence of several age-related diseases such as cardiovascular disease (CVD), cancer, and diabetes by modulating a few key processes such as oxidative stress and autophagy [1]. Reduced oxidative stress can be achieved by several different mechanisms: these include a reduction in the rate at which oxygen free-radials are generated, an increase in the rate at which such radical oxygen species (ROS) are detoxified, and hence a reduction in the damage they cause at the principal macromolecule. Caloric restriction (CR) decreases [2] age-related mitochondrial ROS production in heart and may increase the bioavailability of NO thus preventing atherogenesis [3–6] and decreases lipid accumulation and consequently lipid peroxidation. Moreover, the oxidative stress is a key player in the development and the progression of liver cirrhosis [7], which is known to be a precursor of hepatocellular carcinoma (HCC). Metabolic syndrome is associated with an increase of the oxidative stress status in the body that, in turn, can favour the chronic inflammation that is at the basis of liver transformation in HCC. Physical activity was associated with lower CVD and overall mortality, even outweighing the negative health effects of overweight [8]. Obesity was recognized as a major risk factor for several common types of cancer, of which pancreatic and liver cancer show the highest increase in risk [9, 10]. Obesity enhances HCC development through lipid accumulation within hepatocytes, thereby leading to a chronic low-grade liver inflammation, involving various cytokines and adipokines. The aim of this study was to combine an* in vivo* model with an* in vitro* model to assess whether overweight affects (i) lipid accumulation, (ii) nitric oxide production, and (iii) the extracellular-signal-regulated kinases, heat shock protein 27 (HSP27) expression in rat cardiomyocytes (H9C2), and hepatocarcinoma (Hepg-2) cell lines. Accordingly, we compared oxidative stress markers of young man overweight-serum before and after low-fat diet and moderate physical exercise.

## 2. Methods

### 2.1. Ethical Approval

The experimental procedures followed the rules approved by the Ethics Committee of the Second University of Naples. In detail, patients were informed of the research and gave permission for the use of serum samples.

### 2.2. Diet and Exercise Intervention

Serum samples for this study were obtained from 20 overweight young men (age range 18–30, mean). All participants were overweight (mean body mass index (BMI) 27 ± 1.5) and metabolically healthy. Once enrolled in the program, participants underwent a complete medical history and physical examination, followed by a 21-day diet and exercise intervention. From dietary analysis software, prepared meals contained 12–15% of calories from fat, 15–20% of calories from protein, and 65–70% of calories from primarily unrefined carbohydrate, high in dietary fiber. Carbohydrates were primarily in the form of high-fiber whole grains (5 servings/day), vegetables (4 servings/day), and fruits (3 servings/day). Protein was from plant sources, nonfat dairy (up to 2 servings/day), and fish/fowl (31/2 oz. portions 1 day/wk and in soups or casseroles 2 days/wk).

Before starting the exercise training, subjects underwent a graded treadmill stress test according to a modified Bruce protocol to determine the appropriate individual level of exercise intensity. On the basis of the results, the subjects were provided with an appropriate training heart rate value and given an individualized walking program including both level and graded walking. The exercise regimen consisted of daily treadmill walking at the training heart rate for 45–60 min.

Twelve-hour fasting blood samples were drawn from the subjects in Vacutainers (Becton-Dickinson Vacutainer Systems) containing SST clot-activating gel between 6:30 and 8:00 AM days (preintervention) and 21 days (postintervention). The blood was transported on ice to the laboratory, and the serum was separated by centrifugation and stored at 80°C until analyzed. Weight was measured by using a scale from Pennsylvania Medical Scales (model no. 7500). Height was measured with a stadiometer from Seca, attached to the wall. BMI was calculated as weight (kg)/height (m^2^). Baseline clinical characteristics of the study population are summarized in Table 1. Laboratory parameters such as AST, ALT, creatinine, urea, total cholesterol, triglycerides, GGT were carried out using standard clinical chemical methods.

### 2.3. Serum Evaluation of Oxidative Stress Markers

#### 2.3.1. Thiobarbituric Acid-Reactive Species (TBARS) Assay

Sera samples were incubated with 0.5 mL of 20% acetic acid, pH 3.5, and 0.5 mL of 0.78% aqueous solution of thiobarbituric acid. After heating at 95°C for 45 minutes, the samples were centrifuged at 4000 r.p.m. for 5 minutes. In the supernatant fractions TBARS were quantified by spectrophotometry at 532 nm [11]. Results were expressed as TBARS *μ*M/*μ*g of serum protein. Each data point is the average of triplicate measurements, with each individual experiment performed in triplicate.

#### 2.3.2. Nitrite Levels

Nitric oxide is rapidly converted into the stable end products nitrite and nitrate. Nitrite was measured by the Griess reaction. Briefly, 10 *μ*L of serum was mixed with an equal volume of Griess reagent (0.5% sulfanilamide, 2.5% H_3_PO_4_, and 0.05% naphthylethylene diamine in H_2_O) and incubated for 10 min at room temperature. Absorbance was assayed at 550 nm and compared with a standard curve obtained using sodium nitrite.

#### 2.3.3. Superoxide Dismutase (SOD) Activity

Activity of superoxide dismutase (SOD) was measured with a superoxide dismutase assay kit (Cayman Chemical, Ann Arbor, MI) according to the manufacturer's protocol. Each data point was performed in triplicate, and the results were reported as mean absorption ± standard deviation.

### 2.4. *In Vitro* Cell Culture Studies

Rat cardiomyocytes (H9C2) (ATCC, Manassas, VA) and human liver hepatocellular carcinoma cells (Hepg2) were cultured in DMEM supplemented with 10% fetal bovine serum, 100 U/mL of penicillin, and 100 lg/mL of streptomycin in 150 cm^2^ tissue culture flasks at 37°C in a humidified atmosphere of 5% CO_2_. The cells were fed every 2-3 days and subcultured once they reached 70–80% confluence. The cells were then incubated for 72 h with media containing 10% of pre- and postintervention low-fat diet subjects sera.

#### 2.4.1. Quantitative Oil Red O Assay

ORO solution was made by mixing 4 mL of ORO stock solution (0.014 g red oil in 4 mL isopropanol) with 1.4 mL of distilled water then undergoing filtering [12]. HepG2 and H9C2 cells were cultured in a 96-well microplate at 5,000 cells/well and treated for 72 h with pre and postintervention sera. After removing the fixative solution (4% formaldheide) from each well of the 96-well culture plate, the cells were washed three times with PBS. A 50 *μ*L of the ORO solution was then added to each well and incubated at room temperature for 2 hours. After removing the ORO solution from each well, the cells were washed with PBS until the solution became clear. After washing and drying completely, 100 *μ*L of extraction solution was then added to each well and incubated for 10 minutes, followed by a gentle vibration to release ORO staining. The extract solution containing ORO released from the cells underwent OD measurement at a wavelength of 520 nm using ELX800 Universal Microplate Reader (Bio-Tek instruments, Inc, Winooski, VT). All the tests were performed in triplicate and the means were calculated as final results.

#### 2.4.2. Western Blots

Cardiac H9C2 and HepG-2 cells were collected by centrifugation and then resuspended in ice-cold 50 mM potassium phosphate buffer (pH 7.4), containing 2 mM EDTA. The cells were sonicated for 10 seconds, followed by centrifugation at 13,000 g for 10 min at 4°C. The resulting supernatants were collected and kept on ice for immediate measurements, as described below. Protein expression was determined by western blot. Briefly, H9C2 and HepG2 cells were cultured with sera for 72 hours, and then cell pellets were lysed with 1 mL of lysis buffer. The lysates were centrifuged at 12,000 rpm for 10 minutes at 4°C. The supernatants were used to detect ERK, pERK, and Hsp27 and 90. All the western blots were repeated for three times. Western blot for tubulin was used as internal control. To quantify the results, the relative amount of each protein was determined.

#### 2.4.3. Superoxide Anion Production

Superoxide anion production in mitochondria was determined by hydroethidine (HE) staining. The treated and untreated cells were incubated for 1 h with 20 ng/mL HE and were scraped and washed twice with PBS and the cell pellet was added to 1 mL PBS. HE-superoxide anion (HE-O) accumulation was measured by FACScan flow cytometer (FACScan, Becton Dickinson) using CellQuest software. For each sample 2 × 10^4^ events were acquired. Analysis was carried out in triplicate in at least three separate experiments.

### 2.5. Statistical Analysis

Statistical analyses were performed with GraphPad Prism version 4.0 for Windows, GraphPad Software (San Diego, CA). Preintervention and postintervention values were compared by matched-pair *t*-tests. All data are expressed as means ± SE unless otherwise noted. A *P* value of < 0.05 was considered statistically significant.

## 3. Results

### 3.1. Evaluation of Serum Biochemical Parameters and Oxidative Stress Markers in Young Overweight Men in Basal Conditions and after Low-Fat Diet

Anthropometric and metabolic data and oxidative/antioxidative status of pre- and postintervention (21 days) sera of men with low-fat diet and moderate physical exercise are presented in Table 1. Intervention significantly reduced body weight and BMI. Interestingly, the values were within the normal range before low-fat diet but they were additionally decreased after the change in diet program and exercise. Total-Chol, LDL-Chol, and HDL-Chol were all significantly reduced (*P* < 0.01 for all). A slight decrease of fasting glucose, ALT, and AST was also recorded but without reaching statistical significance whereas the TG and LDH levels did not significantly change. The decrease in serum thiobarbituric acid reactive substance (TBARS), a marker of liver lipid peroxidation, paralleled by an increase in nitric oxide (NO) value and SOD activity in postintervention (*P* < 0.05) subjects, was also observed. The latter finding could be related to an antioxidant activity of high vegetable intake during the energy-restricted diet intervention. Additionally, the diet had no adverse effects on Hb, MCV, and MCH levels (data not shown).

### 3.2. Effect on Survival, Oxidative Stress, and Neutral Lipid Accumulation in H9C2 Myocardiocytes

**Figure 1:**
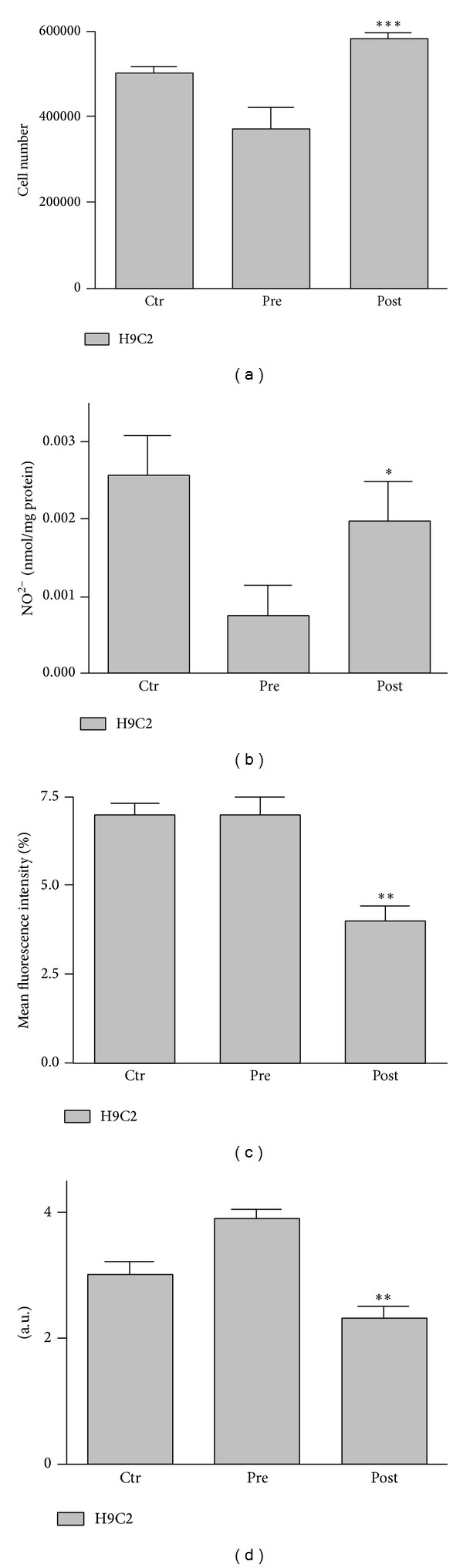
Effect of overweight young man sera on cell number, oxidative stress, and neutral lipid accumulation in H9C2 cardiocyte cell line. (a) H9C2 cell number exposed for 72 h to pre- and postintervention sera. (b) NO levels assessed by Griess method in the medium of H9C2 cells exposed for 72 h to pre- and postintervention sera. (c) Mitochondrial superoxide anion production in H9C2 cells exposed for 72 h to pre- and postintervention sera was analysed by HE (20 ng/mL) staining. Dye accumulation was analysed with FACScan flow cytometer (FACScan, Becton Dickinson) by the CellQuest software. For each sample, 2 × 10^4^ events were acquired. (d) Neutral lipid accumulation evaluated by ORO-based colorimetric assay in H9C2 cells exposed for 72 h to pre- and postintervention sera. Control (Ctr) was assumed to be cells cultured with 10% fetal bovine serum. All analyses were carried out in triplicate from at least three separate experiments. Values, means. Bars, SDs. ∗ denotes *P* < 0.05, ∗∗ denotes *P* < 0.003, and ∗∗∗ denotes *P* < 0.001.

**Figure 2:**
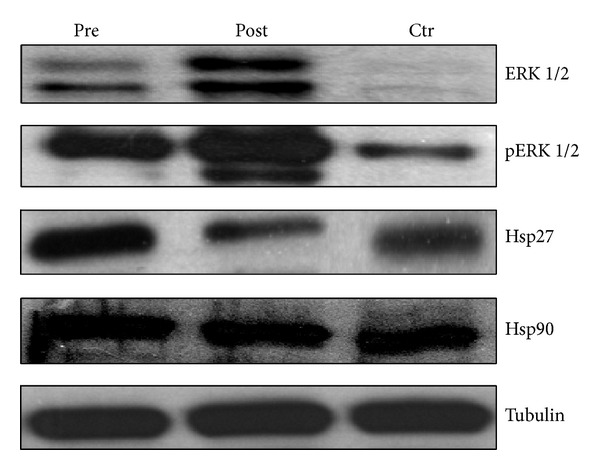
Effects of overweight young man sera on total ERK1/2, phosphorylated ERK1/2 (p-ERK1/2), Hsp27, Hsp90, and tubulin expression evaluated by western blotting analysis in H9C2 cells. H9C2 cells were treated for 72 h with either 10% fetal bovine serum (Ctr) or pre- (pre) or postintervention (post) sera. The bands associated with ERK1/2, phosphorylated ERK1/2 (p-ERK), Hsp27, Hsp90, and house-keeping tubulin expression were opportunely visualized as explained in Section 2.4.2. The expression of the house-keeping protein tubulin was used as loading control. The experiments were repeated several times and gave always similar results.

In order to identify if 3 weeks of caloric restriction combined with moderate exercise could affect cardiocyte survival, we cultured H9C2 cells for 72 h with pre- and postintervention sera collected from subjects (10%). The postintervention sera significantly increased (*P* = 0.0011) H9C2 cell number (Figure 1(a)) compared to the preintervention sera-treated cells. The increase of cell number in the postintervention sera-treated cells was paralleled by an increase of NO due to decreased mitochondrial superoxide anion production (Figures 1(b) and 1(c)) (*P* = 0.0140). Moreover, the addition of overweight young subjects sera for 72 h induced neutral lipids accumulation (*P* = 0.0026) in H9C2 cells as evaluated by Oil Red O (ORO) assay (Figure 1(d)). To additionally determine the effects of pre- and postintervention sera-induced H9C2 cell proliferation, we evaluated the expression of several modulators involved in the regulation of both cell proliferation and oxidative stress. We found that treatment for 72 h of H9C2 cells with postintervention sera significantly increased the activity of ERK and decreased Hsp27 expression if compared to preintervention sera-treated cells as shown in Figure 2.

### 3.3. Overweight Young Subjects Sera Stimulate HepG2 Cell Proliferation and Atherogenic Lipid Particle Production and Secretion

**Figure 3:**
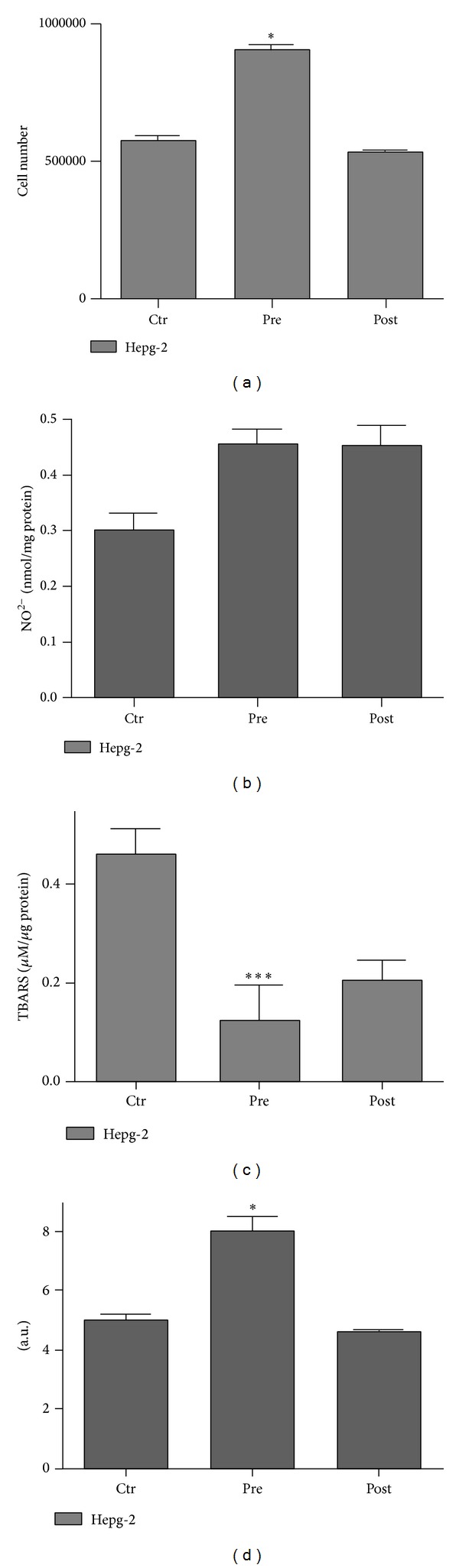
Effect of overweight young man sera on proliferation, oxidative stress, and neutral lipid accumulation in HepG2 cell line. (a) Cell number of HepG-2 exposed for 72 h to pre- and postintervention sera; (b) NO level assessed by Griess method in the medium of HepG-2 cells exposed for 72 h to pre- and postintervention sera. (c) Lipid peroxidation levels assessed using the TBARS method in HepG-2 cells exposed for 72 h to pre- and postintervention sera. (d) Neutral lipid accumulation evaluated by ORO-based colorimetric assay in HepG-2 cells exposed for 72 h to pre- and postintervention sera. Control (Ctr) was assumed to be cells cultured with 10% fetal bovine serum. All analyses were carried out in triplicate from at least three separate experiments. Values, means. Bars, SDs. ∗ denotes *P* < 0.05, ∗∗ denotes *P* < 0.003, and ∗∗∗ denotes *P* < 0.001.

**Figure 4:**
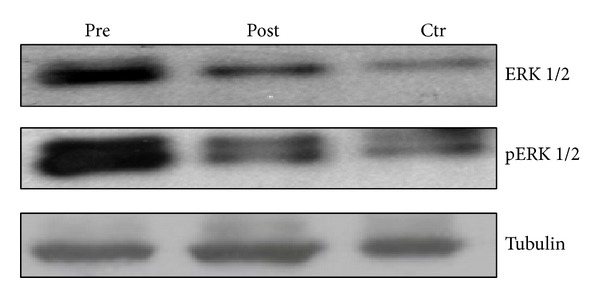
Effects of overweight young man sera on total ERK1/2, phosphorylated ERK1/2 (p-ERK1/2), and tubulin expression evaluated by western blotting analysis in HepG2 cells. HepG2 cells were treated for 72 h with either 10% fetal bovine serum (Ctr) or pre- (pre) or postintervention (post) sera. The bands associated with ERK1/2, phosphorylated ERK1/2 (p-ERK), and house-keeping tubulin expression were opportunely visualized as explained in Section 2.4.2. The expression of the house-keeping protein tubulin was used as loading control. The experiments were repeated several times and gave always similar results.

To additionally investigate whether 3 weeks of low-fat diet and moderate exercise could modulate HCC cell proliferation and alter their lipid metabolism, we treated HepG-2 cells for 72 h with pre- (pre-HepG-2) and postintervention sera (post-HepG-2). The postintervention sera-treated HepG-2 cell number (Figure 3(a)) decreased of about 50% compared to preintervention sera-treated cells (*P* < 0.03). Moreover, the amount of both total and activated isoforms of ERK-1/2 significantly decreased when compared to the preintervention sera-treated HepG-2 cells (Figure 4). We also compared the lipoprotein and lipids secreted by HepG2 cells after 72 h of incubation with pre- and postintervention sera. The results showed a decrease in cholesterol, LDL, secreted VLDL, and hepatic lipase (HL) enzyme activity in post-HepG-2 media when compared to pre-HepG-2 cells (Table 2). However, there was no significant change in HDL, AST, and ALT levels. These results demonstrate that 3 weeks of caloric restriction combined with moderate exercise were able to decrease HCC cell proliferation and atherogenic lipid production. Therefore, we performed ORO assay on pre- and postintervention sera-treated HepG2 cells. The results showed a clear decrease in hepatocyte neutral lipid content in postintervention sera-treated HepG2 cells versus preintervention sera-treated HepG2 cells (Figure 3(d)).

## 4. Discussion 

The main purpose of this exploratory study was to demonstrate that 3 weeks of low-fat diet associated with moderate physical activity of young overweight men may counteract cardiocyte and hepatocarcinoma oxidative stress and modulate cell proliferation caused by overweight. The subjects enrolled (age 24 ± 6 years) in this study were metabolically healthy with biochemical parameters (Table 1) in the normal range. We have found elevated serum lipid peroxidation levels and a reduction in the bioavailability of nitric oxide (NO) due probably to a decrease of superoxide dismutase activity. Recently, authors identified that oxidative stress status is an additional contributing mechanism for increased cardiovascular (CV) risk factors associated with CV disease in obese subjects [13, 14]. It is noteworthy that one of the well-known effects of lipid peroxidation is LDL increase that is, in turn, involved in the formation of early atherosclerotic lesions, according to oxidative theory for atherosclerosis [15]. The intervention (3 weeks of low-fat diet and moderate physical activity) significantly reduced Total-Chol, LDL-Chol, and HDL-Chol serum concentration and lipid peroxidation (TBARS value) of about 2-fold when compared to preintervention sera. Overweight young men sera showed low nitric oxide (NO) concentration that reached an about 3-fold increase in the same subjects after 3 weeks of low-fat diet and physical activity. NO increase could be due to the elevation of scavenger antioxidant enzyme, superoxide dismutase (SOD), suggesting a potential involvement of SOD in regulating the balance between NO and peroxynitrite. Overweight status can trigger a cascade of events, increasing cardiocyte cytotoxicity through alterations in circulating signaling molecule levels. We investigated the effect of pre- and postintervention sera of overweight young man on oxidative status, neutral lipid accumulation, and proliferation of* in vitro* rat cardiocytes (H9C2). NO bioavailability within cardiocytes is likely to be limited to a local environment by both high cytoplasmic concentration of myoglobin (that has high affinity for NO and acts as NO scavenger) and, particularly in diseases, by an abundance of superoxide anions, which can react with NO and, in turn, limit its bioavailability [16]. The preintervention sera treatment of H9C2 cells induced significantly higher levels of mitochondrial superoxide anion that can transform NO into peroxynitrite decreasing its bioavailability. Authors reported that, in Chinese hamster ovary (CHO) cells and in H9C2, the lipid overload induces intracellular accumulation of reactive oxygen species, which subsequently induce endoplasmic reticulum (ER) stress, increase of protein-folding chaperones, and cell death [17]. ERK signaling is activated in both prosurvival and proapoptotic conditions [18]. We observed that prolonged incubation (72 h) of H9C2 with postintervention sera significantly increases their cell number (*P* = 0.0002) when compared to preintervention treated H9C2 cells, and this effect occurred together with increased phosphorylation (and therefore activity) of extracellular-signal-regulated kinases 1 and 2 (pERK1/2). When H9C2 cells were grown with preintervention sera, their cell number was reduced but this effect occurred together with an increase in their protein concentration (data not shown), suggesting an enhancement of protein synthesis leading to cell hypertrophy. Our study presents the evidence that the overweight young man sera induced cytotoxic effects together with increase of neutral lipid accumulation and heat shock protein 27 expression. This small heat shock protein has recently emerged as a critical factor for the protection of cells against many insults, through multiple functions including chaperone activity, mRNA stabilization, maintenance of cytoskeletal architecture, control of redox homeostasis, and apoptosis.

Lifestyle is a pivotal factor in determining the development of cardiovascular and metabolic diseases [19]. Healthful dietary patterns associated with physical activity in young men are important for prevention of risk factors related to CVD and liver diseases (such as nonalcoholic steatohepatitis or NASH) [20]. Overnutrition is thought to lead to excessive accumulation of neutral lipids in the liver, followed by a cascade of prooxidative, hepatotoxic events that increase the risk factor for hepatocellular carcinoma (HCC). We observed that prolonged treatment (72 h) of HepG-2 cells with overweight young man sera increased HepG2 cell number, neutral lipid accumulation, and pERK expression. These effects were counteracted by prolonged treatment of HepG-2 with sera from postintervention subjects, suggesting that all these events occurred with a similar mechanism of action. The precise mechanism by which overweight young man sera induced ERK-activation remains, however, to be elucidated.

In conclusion, our findings suggest that overweight in young man without metabolic syndrome is associated with increased risks of cardiovascular and metabolic disease. Moreover, change of lifestyle in young men can induce beneficial effects on health through changes in lipid metabolism and oxidative stress status.

## Figures and Tables

**Table 1:** Anthropometric and metabolic parameters and oxidative/antioxidative status of young overweight men (*n* = 20), undergoing a 21-day diet and exercise intervention.

	Pre	Post	*P* value
Body weight	100 ± 10	90 ± 2	0.0003
BMI	27 ± 1.5	25 ± 1.8	0.0001
Total-C, mg/dL	200 ± 10	159 ± 7	0.0001
LDL-C, mg/dL	109 ± 5	84 ± 4	0.0001
HDL-C, mg/dL	44 ± 3	39 ± 5	0.0005
TG, mg/dL	110 ± 5	111 ± 7	—
Blood glucose, mg/dL	100 ± 5	92 ± 9	0.0013
AST-GOT	23 ± 2	17.5 ± 0.5	0.0001
ALT-GPT	28 ± 0.5	19.1 ± 0.3	0.0001
LDH	305 ± 5	300 ± 8	0.0230
TBARS *μ*M/*μ*g protein	0.0017	0.00076	0.0156
NO nmol/*μ*g protein	0.00048	0.00073	0.0052
SOD activity U/*μ*g protein	0.0009	0.0014	0.0465

All data are expressed as means ± SE (n = 20 subjects). BMI: body mass index; Total-C: total cholesterol; LDL-C: low-density lipoprotein cholesterol; HDL-C: high-density lipoprotein cholesterol; TBARS: thiobarbituric acid reactive substance; NO: nitric oxide; SOD: superoxide dismutase. *P* value was evaluated according to “Pre” versus “Post” intervention.

**Table 2:** Analyses of biochemical parameters of HepG-2 cells treated for 72 h with pre and postintervention sera.

	Pre	Post	*P* value
Hepatic lipase (U/L)	7.6 ± 0.9	4.1 ± 0.5	0.0042
Triglyceride (mmol/L)	9.2 ± 1	11.1 ± 0.9	0.0707
Total cholesterol (mmol/L)	16.3 ± 2	12.4 ± 1.5	0.0540
HDL-C (mmol/L)	7.7 ± 0.5	7.6 ± 0.7	—
LDL-C (mmol/L)	7 ± 1	3 ± 0.5	0.0034
Glucose (mmol/L)	101 ± 5	93 ± 6	0.1507
AST (U/L)	8 ± 0.5	8.5 ± 0.6	—
ALT (U/L)	1.4 ± 0.2	1.5 ± 0.4	—

## Abbreviations

BMI: Body mass index
CVD: Cardiovascular disease
HDL: High-density lipoprotein cholesterol
LDL: Low-density lipoprotein cholesterol
HSP27: Heat shock protein 27
ERK1/2: Extracellular-signal-regulated kinases 1 and 2
ROS: Radical oxygen species
TBARS: Thiobarbituric acid reactive substance
NO: Nitric oxide
ORO: Oil Red O.
